# Superselective superior rectal artery embolization in the treatment of hemorrhoidal disease

**DOI:** 10.3389/fmed.2025.1530981

**Published:** 2025-02-26

**Authors:** Tao Jiang, Lichao Fan, Xuesong Tang, Zhigang Xu, Wenjiang Wu

**Affiliations:** Department of Proctology, Shenzhen Hospital (Futian) of Guangzhou University of Chinese Medicine, Shenzhen, China

**Keywords:** superior rectal artery embolization, hemorrhoidal disease, endovascular intervention, minimally invasive procedure, hemorrhoidal bleeding

## Abstract

Hemorrhoids are a prevalent and benign anal disorder for which minimally invasive treatments are increasingly preferred. The UK National Institute for Health and Care Excellence clinical guideline (2010) recommends hemorrhoidal artery ligation as a treatment option for hemorrhoidal disease. Superior rectal artery embolization (SRAE) leverages this principle by using digital subtraction angiography to precisely identify and superselectively embolize the arteries supplying the hemorrhoidal region. This procedure has demonstrated favorable clinical outcomes. SRAE is minimally invasive, painless, preserves the anal sphincter and normal anal anatomy, and offers a quick recovery, establishing it as an effective minimally invasive surgical option. As a result, this technique has gained increasing clinical recognition and adoption. This article examines the efficacy and safety of superselective SRAE for hemorrhoidal disease.

## 1 Introduction

Hemorrhoidal disease is a common benign anal disorder, with one of its primary clinical symptoms being rectal bleeding, which, in severe cases, can lead to anemia and significantly reduce a patient’s quality of life (QOL). The prevalence of hemorrhoidal disease ranges from 4% to 35% (1). It is characterized by the dilation of the hemorrhoidal venous plexus and requires clinical treatment in symptomatic patients (2). Internal hemorrhoids are classified into four stages according to the Goligher classification (3). Recent research indicates a trend toward minimally invasive treatment methods for hemorrhoids. The UK National Institute for Health and Care Excellence 2010 clinical guideline recommends hemorrhoidal artery ligation, based on the principle of vascular dissection, as a safe alternative to traditional hemorrhoidectomy (4). Additionally, Vidal et al. (5) were the first to propose embolization of the superior rectal artery (SRA) using coils, demonstrating that this technique is feasible, safe, and well-tolerated.

Superior rectal artery embolization (SRAE), also referred to as the emborrhoid technique, is a minimally invasive, image-guided procedure for treating hemorrhagic hemorrhoidal disease, with efficacy and recurrence rates comparable to those of stapled hemorrhoidopexy (6). Digital subtraction angiography (DSA) is globally recognized as the “gold standard” for vascular imaging, and using DSA for endovascular embolization offers significant advantages over Doppler-guided hemorrhoidal artery ligation. These advantages include clear visualization of the hemorrhoidal blood supply and its branches, precise localization, and permanent occlusion of the hemorrhoidal arteries using endovascular coils. This innovative approach provides a minimally invasive solution for managing hemorrhoid-related bleeding disorders (7, 8). Given its simplicity, SRAE can be broadly implemented in hospitals equipped with vascular intervention centers. This article reviews the efficacy and safety of superselective SRAE for the treatment of hemorrhoidal disease.

## 2 Pathogenesis of hemorrhoidal disease and vascular pathophysiology

Understanding the anatomy of the anorectum and the vascular branching pattern is crucial for effectively managing rectal and hemorrhoidal bleeding disorders and providing optimal treatment options. Recent studies have shown that the development of hemorrhoidal disease is closely associated with hyperperfusion and neovascularization in the arteries supplying the affected area. Hemorrhoidal disease is characterized by localized masses formed from the pathological thickening, hypertrophy, or migration of the anal cushion (9). Internal hemorrhoids, in particular, result from the dilation and tortuosity of the complex venous plexus (10). The dilation of the internal hemorrhoidal venous plexus is primarily attributed to increased arterial blood flow from the SRA to the anal cushion (8). The development of the anal cushion is influenced by the arterial supply to the rectal corpus cavernosum (CCR) (11), which is mainly provided by the terminal branches of the SRA. The SRA, the primary artery supplying internal hemorrhoids, divides into three to five branches (12). Additionally, the lower rectum and anal canal receive blood from the middle rectal artery and the inferior rectal artery (IRA), which, along with the SRA, supply blood to the rectum. Embolizing the distal branches of the SRA with embolic agents effectively reduces blood flow to the anal cushion and controls hemorrhoidal bleeding. Even if all visible branches of the SRA are occluded, this procedure does not cause ischemic complications (8).

Angiography of the SRA is an advanced technique that enables precise assessment and localization of hemorrhoidal artery branches. It provides visualization throughout the entire process of endovascular interventional embolization, facilitating superselective embolization of the arteries supplying the hemorrhoidal region, and has demonstrated strong clinical efficacy (13, 14). This minimally invasive and painless procedure preserves normal anal anatomy, including the anal sphincter, and allows for rapid postoperative recovery (15), reduced length of hospitalization (16) making it a highly effective minimally invasive surgical option. Consequently, this technique has gained increasing recognition and widespread clinical use.

## 3 Effectiveness of SRAE in the treatment of hemorrhoidal disease

### 3.1 Bleeding

Bleeding symptoms are primarily assessed using the French bleeding score, which ranges from 0 (no bleeding) to 9 (daily bleeding requiring blood transfusion). The score evaluates four aspects: frequency of bleeding, blood flow, whether anemia is present, and the severity of symptoms (17). The scoring system is simple and can be easily repeated to monitor changes in bleeding. Currently, SRAE is still being explored as a treatment for hemorrhagic hemorrhoidal disease, and findings from various studies have shown inconsistent results. A reduction of at least 2 points on the French bleeding score is generally considered a criterion for successful treatment. According to Vidal et al. (5, 8), 28% of patients did not experience an improvement in bleeding symptoms and required one or more additional embolization procedures. Luo et al. (18) treated 15 patients with grade II–IV hemorrhoidal disease using SRAE, achieving successful interventions in all cases. During a follow-up period of 19.0 ± 2.6 months, all patients experienced resolution of bleeding symptoms without serious complications. Tradi et al. (19) performed SRAE on 25 patients with grade II and III hemorrhoids. Twelve months post embolization, 18 patients (72%) achieved clinical success, with their bleeding scores decreasing from 5.5 to 2.3 (*p* < 0.01). Moggia et al. (20) performed SRAE on 16 patients with chronic bleeding hemorrhoidal disease, including those who had contraindications to conventional surgery (e.g., coagulation disorders, ongoing anticoagulant or antiplatelet therapy, or inability to assume the necessary surgical position). At the 12-month follow-up, no postoperative or short-term complications were observed, and 14 patients (87.5%) reported reduced bleeding symptoms and an improved QOL. Han et al. (13) retrospectively analyzed 32 patients with grade II and III internal hemorrhoids who had chronic rectal bleeding and contraindications to surgery or declined conventional hemorrhoidectomy. After SRAE, 27 patients (84.4%) experienced symptom relief, with a follow-up duration of at least 1 year and no significant complications. Four patients (14.8%) experienced a recurrence of bleeding within the first few months of follow-up. These patients were successfully managed with internal iliac arteriography and branch embolization, with no further bleeding episodes observed over at least 3 months of follow-up. Potential reasons for treatment failure or recurrence may include arteriovenous malformations in the CCR, multiple collateral vascular anastomoses between the distal branches of the SRA and the middle and inferior rectal arteries, blood flow reconnection, and coagulation disorders occurring during coil embolization (8, 15). Given the current literature, more studies are needed to thoroughly evaluate the efficacy of SRAE for managing hemorrhoidal disease.

### 3.2 Prolapse

The Goligher classification system is used to evaluate the severity of internal hemorrhoid prolapse. The emborrhoid technique has demonstrated the ability to reduce hemorrhoid size while preserving anal sphincter function within 1 month of treatment (9, 19, 20). However, there is still insufficient evidence to show consistent improvement in the Goligher classification (17). Moussa et al. (8) performed embolization on 30 patients with hemorrhoidal disease, but results indicated no change in the severity of prolapse as measured using the Goligher classification. This suggests that conventional surgery is necessary for prolapse treatment. Tradi et al. (19) found that the emborrhoid technique alleviated symptoms of stage II and III internal hemorrhoidal prolapse, likely due to a reduction in arterial blood flow and subsequent decrease in hemorrhoidal congestion. However, for stage IV hemorrhoids, surgical excision remains the recommended treatment. Eberspacher et al. (21) described a case involving a 58-year-old male patient with a history of internal hemorrhoidal prolapse. The patient underwent hemorrhoidal artery embolization. However, after three endoscopic balloon dilations (performed at 2 months, 2 months and 3 weeks, and 3 months post embolization), the prolapse did not improve. After hemorrhoidectomy, there was no prolapse at the 6-month follow-up. The study emphasized the importance of carefully considering the benefits and limitations of the emborrhoid technique before proceeding with surgical intervention. Zakharchenko et al. (22) observed a significant decrease in the size of internal hemorrhoids 1 month after embolization, with an average reduction of 43%, particularly in stages I–III. This study suggested that patients with lower-stage hemorrhoids were more responsive to treatment. Han et al. (13) retrospectively analyzed 32 cases of hemorrhoidal disease treated with SRAE, reporting a marked reduction in prolapse in 27 patients. Sun et al. (23) presented a case of chronic hemorrhoidal bleeding treated with embolization of both the superior and inferior rectal arteries. At 1 month post treatment, the hemorrhoidal masses had decreased in size (from 1.9 cm to 1.2 cm), blood flow had significantly decreased, and both internal and external sphincter contractions were normal. Komekami et al. (24) documented a case in which a patient with rectal arteriovenous malformations associated with hemorrhagic hemorrhoids showed reduced internal hemorrhoid size 6 months post embolization, as confirmed via colonoscopic examination. The reduction in hemorrhoid size was thought to result from the closure of internal hemorrhoidal varices without reflux following embolization, leading to decreased blood flow in the hemorrhoidal venous plexus and subsequent shrinkage of the hemorrhoidal masses (25).

### 3.3 QOL

Hemorrhoidal disease is clinically manifested by symptoms such as bleeding and prolapse, often accompanied by anal itching and pain. Recurrent episodes of these symptoms can significantly impact patients’ QOL (26, 27). QOL is assessed using a scoring system ranging from 1 (no discomfort) to 5 (permanent discomfort). Tradi et al. (19) reported that 72% of patients experienced improved QOL at 12 months post embolization. Moggia et al. (20) found that following embolization of 16 patients, 87.5% experienced an improvement in their QOL scores from 4 to 2. Similarly, Moussa et al. (17) retrospectively analyzed 45 patients who underwent the emborrhoid procedure and found that at the 1-year follow-up, QOL scores improved from 3.5 to 2. The primary reasons for this improvement were a reduction in bleeding and a decrease in the size of the hemorrhoidal masses.

### 3.4 Pain

A major reason many patients with hemorrhoidal disease decline traditional surgery is the fear of postoperative wound pain and the long recovery period, which can interfere with returning to work and daily activities. The SRAE technique addresses these concerns by offering a painless alternative, as it does not involve direct damage to the anorectal area and can be performed as an outpatient procedure (8). Pain is commonly assessed using the visual analog scale, which is widely recognized by experts (28). Several studies (16, 20, 22, 26) have demonstrated that the SRAE technique provides effective short-term relief for bleeding internal hemorrhoids, with no reported complications such as pain and no need for postoperative pain medication. However, Han et al. (13) reported that 12.5% of patients experienced mild to moderate pain, which resolved on its own without requiring additional treatment.

## 4 Choice of embolic agents

Various embolic agents are used in the SRAE technique, each yielding different results (13). The effectiveness of these agents depends on the extent of the blood supply area blocked and the degree of vessel occlusion. Commonly used embolic agents include spring coils, gelatin sponge particles, and polyvinyl alcohol (PVA) particles. Several studies (14, 16, 20) have demonstrated that spring coil embolization of the SRA is technically feasible, safe, and well-tolerated. In a prospective study by Tradi et al. (29), which compared multiple embolic agents in a healthy porcine model, microspheres were found to have fewer complications than liquid embolizers and were able to occlude farther than microcoils. The study concluded that microsphere embolic agents are more effective while maintaining the same safety profile as spring coils. Vidal et al. (5) emphasized that spring coil embolization should be as thorough as possible to prevent rebleeding. Zakharchenko et al. (22) reported that using a combination of pellets and coils for SRA embolization does not cause ischemic symptoms and is effective. The hemostasis rate for gelatin sponge pellet embolization was 84.4% (13), lower than that of 92.5% achieved with coils combined with PVA pellet embolization (22) but higher than that of 72% achieved with coil embolization alone (30). Conversely, another study (17) indicated no significant difference in clinical success rates between using pellets with spring coils versus spring coils alone, with a success rate of 68%. The use of alternative embolization materials, such as liquid embolic agents, may yield better outcomes. Huang et al. (31) conducted a study involving 27 patients with acute hemodynamic instability from lower gastrointestinal bleeding who underwent N-butyl cyanoacrylate (NBCA) microcatheterization. Bleeding stopped immediately in all patients. In four patients, recurrent bleeding was observed at different times after embolization. Kickuth el al (32). retrospectively reviewed 20 patients who had undergone diagnostic angiography and superselective microcatheter embolization to control acute LGI hemorrhage superselective microcatheter embolization to control acute LGI hemorrhage, 14 patients he last treatment was in a time span of 5 years. The results suggested that NBCA embolization could be a safe alternative for treating lower gastrointestinal bleeding, although further clinical trials are necessary.

## 5 Indications and contraindications

Superselective SRAE is particularly well-suited for patients who decline traditional surgical options or for those with bleeding-dominant internal hemorrhoids of grades II and III who are unable to tolerate conventional surgery due to underlying conditions such as cardiovascular disease, blood disorders, diabetes mellitus, or inflammatory bowel disease. It is also an option for patients who have experienced a recurrence after previous surgery. Currently, no studies have identified specific contraindications for using this method to treat internal hemorrhoids. However, it is not recommended for patients with grade IV internal hemorrhoids, external hemorrhoids or thrombosed hemorrhoidal disease (14), or contraindications to interventional procedures.

## 6 Complications

Recent studies (12, 13) have shown that no significant complications have been observed following embolization, with no cases of local ischemia or inflammatory complications reported. Only a very small number of patients experienced mild pain or bleeding discomfort, which resolved on its own without the need for medication (17, 22). Sun et al. (9) found that among 23 patients with hemorrhagic internal hemorrhoids who underwent embolization of the IRA, 34.78% experienced a sense of urgency during the procedure. Additionally, embolization of the IRA branches was less effective for patients with contraindications to interventional procedures. When IRA branches were embolized simultaneously, the likelihood of acute, severe sensations increased, but these symptoms typically resolved within 1–3 days postoperatively. Furthermore, 17.4% of patients experienced minor bleeding within 3–6 days after the procedure. This technique also allows for repeated embolization, which may enhance treatment effectiveness for some patients (8, 17, 21).

## 7 Conclusion

Several clinical studies (33–38) have reported short-term efficacy rates of approximately 90% and long-term efficacy rates of 70–92% in small-scale studies, with follow-up periods of 6–46 months. Minimally invasive treatments for hemorrhoidal disease are becoming increasingly popular, and numerous studies have confirmed that the SRAE technique is both feasible and a safe alternative. Nevertheless, larger and more comprehensive studies with longer follow-up periods are necessary to better understand the efficacy, recurrence rates, and potential complications of the procedure. Given the high levels of patient satisfaction and improvement in hemorrhoidal symptoms, further prospective and multicenter studies are warranted.
